# 4160 Evaluating Student Team Dynamics

**DOI:** 10.1017/cts.2020.210

**Published:** 2020-07-29

**Authors:** Celia Chao, Emma Tumilty, Celia Chao, Judith Aronson, Jonathan D. Hommel, Mark R. Hellmich

**Affiliations:** 1 University of Texas Medical Branch

## Abstract

OBJECTIVES/GOALS: We aimed to explore the students’ assessments of workload distribution by comparing personal reflective commentaries and team documents defining division of labor in a team science setting. METHODS/STUDY POPULATION: The Interprofessional Research Design course models the team science experience by bringing together MD and PhD students to write a research grant. Four teams of 13 students were tasked with both individual and team-based assignments: 1) Each week, each student reported their perception of their own and their team members’ effort over the week (totalling 100%). 2) Iterative work contracts for each team were submitted at four time-points; assigned work toward project completion totalled 100%. 3) Lastly, each student submitted a short commentary reflecting on the prior week’s team dynamics and teamwork. We retrospectively performed a mixed-methods analysis of the workload data. RESULTS/ANTICIPATED RESULTS: Group-reporting in the team contracts remained static throughout the course, often stating equal distribution of workload, whereas individual reporting was more dynamic. Of 13 students, 8 rated more than 50% of the weeks as balanced. Among some students, there was a discordance of workload distribution when comparing the group document to the individual perceptions of work performed by their teammates. Reflective writing mapped more closely to individual quantitative reports. The data also revealed within team variations, where one student may report a higher proportion of their contributions, while the rest of the team attributed that student a lower percentage of the total work. DISCUSSION/SIGNIFICANCE OF IMPACT: An important aspect of team function is workload distribution. Group-based workload discussions may be a useful framework, but does not provide insight into team dynamics, whereas individually reported workload distributions and short reflections seem to more accurately inform us on team function.

